# Radiolucent Matrix Stones in a Transplanted Kidney: A Case Report

**DOI:** 10.7759/cureus.38280

**Published:** 2023-04-29

**Authors:** Mohammed Zain Ulabedin Adhoni, Amy Nagle, Zubeir Ali

**Affiliations:** 1 Urology, The Royal London Hospital, London, GBR

**Keywords:** colloid calculi, matrix stone, radiolucent kidney stone, kidney transplant recipient, endo urology, renal stone surgery, renal stone disease

## Abstract

Matrix stones are a rare form of kidney stones, which are composed of mucoproteinaceous material. They are often difficult to diagnose as they are characteristically radiolucent on CT urinary tract. This difficulty is compounded in transplanted kidneys as obstructing stones commonly present without pain and can cause acute kidney injury. Here, we present a case of a 61-year-old female with a live-donor kidney transplant, who was found to develop deranged renal function on routine follow-up investigations. Therefore, a CT urogram was performed and it showed filling defects in the renal pelvis and upper ureter of the transplanted kidney. Therefore, diagnostic ureterorenoscopy was performed and three stones of about 7-8 mm each were found in the renal pelvis, they were treated by Holmium:Yttrium aluminium garnet (YAG) laser fragmentation. This case report describes the challenges in the management of this rare stone in a transplanted kidney.

## Introduction

Kidney stones are uncommon in transplanted kidneys with an incidence of 1% and a mean duration of 28 months after transplantation. The most common type of kidney stone in a transplanted kidney is calcium-based stones, followed by struvite stones and subsequently uric acid stones [1]. The formation of kidney stones in transplanted kidneys is multi-factorial; however, the most common risk factors are hyperparathyroidism, hypercalciuria, recurrent urinary tract infection, and hypocitraturia [2]. It is a challenge to diagnose stones in transplanted kidneys as obstructing stones often present without pain [3], and can cause acute kidney injury (AKI) in the transplanted kidney [1]. The present case shows a matrix stone that was radiolucent and showed up as a filling defect on a CT urogram making it a diagnostic challenge.

## Case presentation

**Figure 1:**
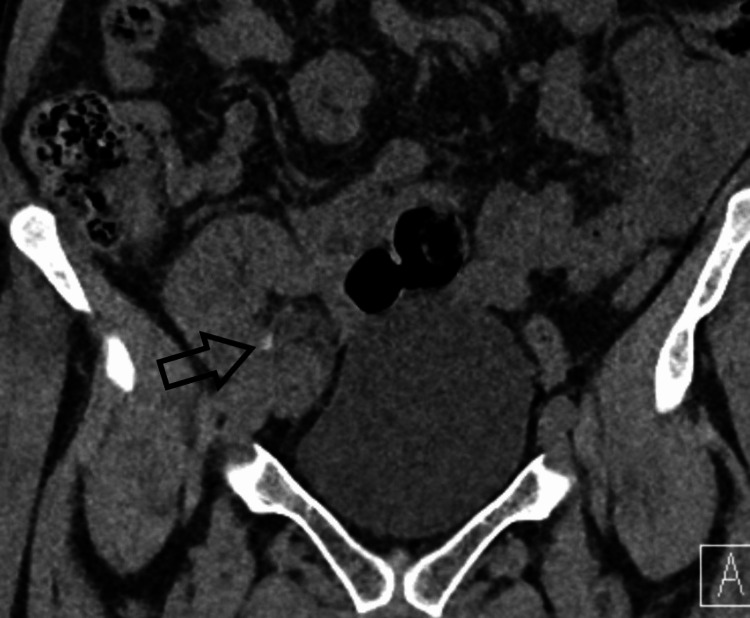
CT non-contrast phase of transplanted kidney showing high-density material in renal pelvis

**Figure 2:**
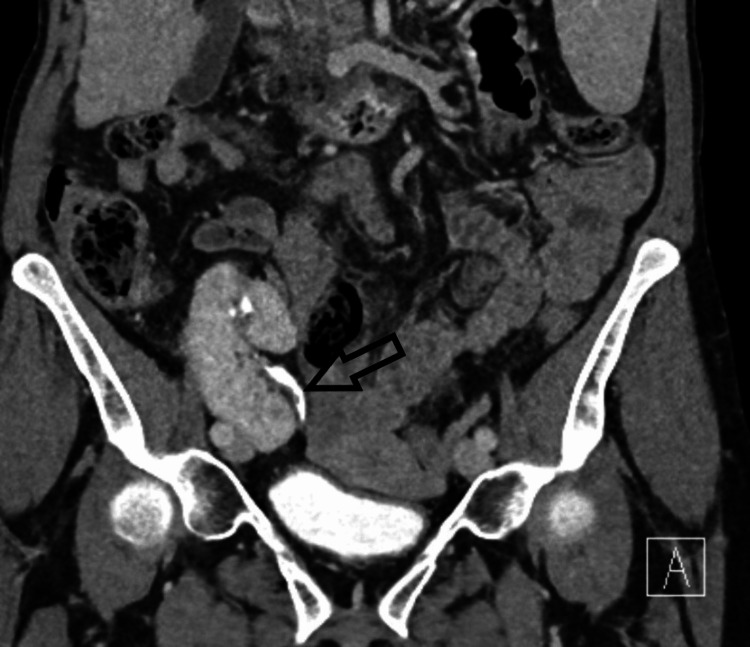
CT urogram of transplanted kidney showing filling defects in the upper ureter

A 61-year-old female with a live donor kidney transplant done in 2018 due to tubulointerstitial nephritis with a medical history of primary biliary cirrhosis, hypersplenism, right middle lobe bronchiectasis was being investigated for painless visible hematuria and worsening renal function of the transplanted kidney in 2020. Her CT urogram showed a 9 mm filling defect in the transplanted kidney ureter in the excretory phase with high-density product within the mid-renal calyces (Figure 1) in the transplanted kidney in the pre-contrast phase. It also showed urothelial irregularity in all calyces, renal pelvis, and ureter, and perinephric stranding (Figure 2).

These findings were discussed in our urology multi-disciplinary team meeting with the outcome being a recommendation for a diagnostic ureterorenoscopy (URS).

**Figure 3:**
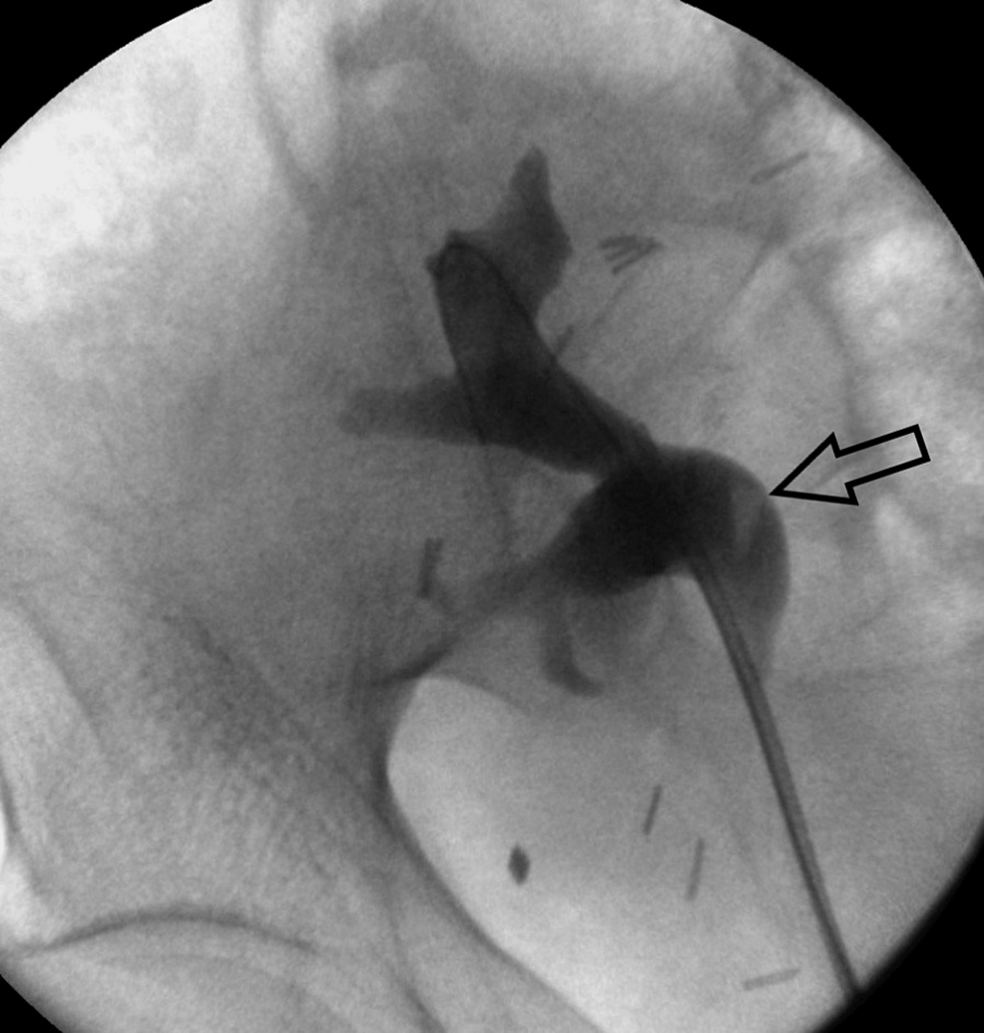
Retrograde pyelogram demonstrated a floating filling defect

The URS was done four months after the CT urogram. The transplanted kidney's ureteric orifice was found at the dome of the bladder and was initially cannulated by a sensor guidewire. Urine was taken for cytology with a ureteric catheter and was negative for malignant cells. Subsequently, retrograde studies were done which demonstrated no urothelial irregularities; however, there were radiolucent mobile masses (floating filling defects) in the pelvis (Figure 3). With the flexible URS, three black stones 7-8 mm in size were visualized in the renal pelvis, and the mucosa of each calyx was normal. The stones were lasered with Holmium:Yttrium aluminium garnet (YAG) and some fragments were basketed for analysis. A final short semi-rigid URS was done to basket out the clots and the remaining fragments, and complete stone clearance was achieved. A 6Fr-10cm JJ stent was inserted into the transplanted kidney.

Her JJ stent was removed via flexible cystoscopy after two weeks. Stone analysis showed no common renal stone constituents and contained protein-matrix material, suggestive of a pure matrix stone. Her metabolic profile (Table 1) and 24-hour urine analysis (Table 2) showed hypocalciuria, hypophosphatemia, and low urinary phosphate. She was stone free in the transplanted kidney on her follow-up non-contrast CT scan in 2022.

**Table 1:** Metabolic profile

	Values	Normal values
Calcium	2.45 mmol/L	2.1-2.6 mmol/L
Adjusted Calcium	2.37 mmol/L	2.1-2.6 mmol/L
Inorganic Phospheros	1.10 mmol/L	1.12-1.45 mmol/L
Urate	283 umol/L	200-430 umol/L

**Table 2:** Urine analysis (24-hour)

	Values	Normal values
Urine Creatinine	2.80 mmol/L	2.5-8.2 mmol/L
Urine Calcium	1.7 mmol/L	
24-hour urine calcium	5.3 mmol/24hrs	2.5-7.5 mmol/24hrs
Urine citrate	0.36 mmol/L	
24-hour urine citrate	1.11 mmol/24hrs	1.3-6.0 mmol/24hrs
Urine citrate:creatinine ratio	0.13 mmol/mmolC	0.11-0.55 mmol/mmolC
24-hour urine phosphate output	14.8 mmol/24hrs	15-50 mmol/24hrs
Urine magnesium	1.15 mmol/L	
24-hour urine magnesium	3.6 mmol/24 hrs	2.4 -6.5 mmol/L
Urine oxalate	<50 umol/L	<50 umol/L
Urine oxalate: creatinine ratio	Unable to calculate.	
Urine protein	0.16 g/L	
Urine protein: creatinine ratio	51.61 mg/mmol	0-30 mg/mmol

## Discussion

Matrix stones are a rare form of radiolucent stones described in the literature for the first time in 1908. They are also known as fibrinomas, colloid calculi, or albumin calculi [4]. They are made of soft mucoproteinaceous material and usually form in the renal collecting system [5]. The risk factors for such stones based on small data sets are female gender, recurrent urinary tract infection, chronic kidney failure, and hemodialysis [6,7]. In a study including 8854 patients who underwent stone analysis, 1.8% of the kidney stones showed non-specific organic material [8]. Matrix stones are characteristically radiolucent and do not have an acoustic shadow on ultrasound; however, on CT scans they may show up as eggshell calcification due to outer calcification and inner soft tissue density [6,7]. A case report characterized a matrix stone on MRI as showing hypointense signal in T1-weighted images and slight hyperintense signal in T2-weighted images with no obvious contrast enhancement after gadolinium administration in T1-weighted images [9].

In a case series done for nine patients with matrix stones, six patients were treated with percutaneous nephrolithotomy (PCNL) in a single session, and three patients had initial URS; however, all of them required either further URS, PCNL, extracorporeal shockwave lithotripsy, or a combination of the aforementioned procedures [6]. In transplanted kidneys, stone clearance with ureterorenoscopy is a viable option with a high rate of stone clearance, especially in experienced centers [10]. We were able to treat a transplanted kidney with three matrix stones of 7-8 mm each, achieving complete clearance with ureterorenoscopy. We believe that it would be the procedure of choice, particularly in patients with uncertain diagnoses.

## Conclusions

Kidney stones are a known complication of kidney transplants. It should be considered if the patient develops AKI as obstructing stones in transplanted kidneys can present without pain, which might add difficulty in diagnosis, particularly if the stone was a matrix stone. Risk factors for matrix stones include female sex, recurrent urinary tract infections, chronic renal failure, and hemodialysis. Matrix stones are radiolucent and present as filling defects on CT urography. We believe that ureterorenoscopy would be the diagnostic tool of choice in cases of uncertainty, in addition to its therapeutic role.
